# On Dislocation of the Crystalline Lens

**Published:** 1842-04

**Authors:** John Walker

**Affiliations:** Surgeon, Manchester, England


*On Dislocation of the Crystalline Lens.* By John Walker, Esq., Surgeon, Manchester, England.—A severe blow, or other injury inflicted on the eye, will sometimes cause a displacement of the lens. This may occur in three different directions; the lens may be thrown backwards into the substance of the vitreous humor, forwards into the anterior chamber, and sometimes forced through the sclerotic coat, when it will be found lying between it and the conjunctiva. Instances of each of these forms of displacement are not unfrequently met with. The lens may also be more partially displaced, either upwards, downwards, or laterally. Dislocation of the lens, however, is not always the result of injury. I have seen cases in which the crystalline had been protruded into the anterior chamber when no accident had occurred, and where the change had been, to all appearance, spontaneous. In one case, a young girl, under the care of my colleague, Mr. Windsor, both eyes were thus affected; the lens was seen lying in the anterior chamber, its transparency but little diminished, of a bright, gold-colored tinge, and very much resembling a lump of clear, almost colorless, jelly. In this instance, the pupils were dilated and immovable, considerable inflammation existed, and the usual concomitants, intolerance of light, pain in the organ, and impaired vision, symptoms which were apparently referable to the irritation caused by the displaced lens. The lenses were extracted, but the mischief engendered had been of too serious a character to be remedied, and the eyes remained perfectly amaurotic. Other instances of spontaneous dislocation are recorded; in some of these, the lens passed either in front or behind the pupil, according to the position of the head, and did not appear to excite much irritation.

It seems most probable that spontaneous dislocation of the lens can only result from a previous thinning or absorption of the capsule, although some of our best writers appear to think that when the lens retains its transparency the capsules remain entire, otherwise they presume that it would become opake, and be absorbed when in contact with the aqueous humor. This, however, does not seem a necessary result, except the texture of the lens were also broken up, which, in cases of spontaneous dislocation, we should not expect it to be.

When the lens is protruded either into the anterior chamber or between the sclerotica and conjunctiva, it will usually be proper to remove it from its new position as early as possible, as it will otherwise be sure to excite inflammation, which may lead to a serious impairment of vision. Indeed, the injury which is sufficient to cause displacement of the lens will very often be productive of other important consequences, as concussion of the retina and severe inflammation of the globe. For the removal of a displaced lens from the anterior chamber, a small incision will alone be requisite; the opening need not extend to more than one-fourth of the circumference of the cornea, when, if it do not readily escape, the introduction of an iris-hook will effect its dislodgement. In one case of dislocation into the anterior chamber, the lens, after extraction, was found completely ossified; it had been several years in its unnatural position, and had either produced or was attended by amaurosis.

If the lens had been forced through the sclerotica, and remain under the conjunctiva, a small incision into the latter tunic will be sufficient for its removal. In one instance which came under my notice, and which was occasioned by a blow, the wound of the sclerotica, through which the lens had been protruded, was scarcely perceptible, the displaced body was easily removed, and was found quite softened and slightly opake; the nature of the case was indicated by the injury, and by the size, shape, and consistence of the foreign body under the conjunctiva, and confirmed on its removal.

If the lens be displaced into the posterior chamber, it had probably better not be interfered with, except it be opake, and its removal seem likely to cause a restoration of vision; but even then, it would be advisable merely to depress it, because the vitreous humor would most likely be disorganized.

It is worthy of remark, that the extraction of a displaced lens from the anterior chamber gave rise to the systematic extraction of cataract by incision of the cornea. Daviel, a French surgeon, took the hint from Petit, who, in 1708, punctured the cornea for the evacuation of a displaced and opake lens.—*N. Y. Lancet.*

